# Bariatric surgery reduces sleep apnea in obese patients with obstructive sleep apnea by increasing pharyngeal cross-sectional area during the early postoperative period

**DOI:** 10.1007/s00405-023-07821-4

**Published:** 2023-01-16

**Authors:** Yuliang Zhao, Tao Li, Guangyuan Zhang, Xiaorong Liang, Yanxia Wang, Jiansheng Kang, Jiangang Ma

**Affiliations:** 1 Department of Otorhinolaryngology, The Second Hospital of Hebei Medical University, Shijiazhuang, 050000 China; 2 Department of General Surgery, The Second Hospital of Hebei Medical University, Shijiazhuang, 050000 China

**Keywords:** Obstructive sleep apnea, Bariatric surgery, Pharyngeal cross-sectional area

## Abstract

**Objective:**

Bariatric surgery (BS) is considered one of the most effective treatments for obese individuals with Obstructive Sleep Apnea (OSA). However, otolaryngologists have raised concerns about the structural alterations caused by BS on the upper respiratory tract, especially, on the pharyngeal cavity.

**Methods:**

In this study, we recruited 42 individuals who underwent BS at our hospital. They were divided into two groups based on apnea–hypopnea index (AHI): mild group (5 ≤ AHI < 15) and moderate-severe group (AHI ≥ 15). The participants were followed up for 12 months and several indicators, including body mass index (BMI), polysomnography (PSG), and acoustic pharyngometry (APh), were assessed repeatedly before surgery and at 3, 6, and 12 months (m) after surgery.

**Results:**

Participants exhibited significant decreases in BMI (*F* = 128.1, *P* = 0.001) and total weight loss (*F* = 176.7, *P* < 0.001) after BS. The AHI value among obese patients with mild OSA decreased significantly within three months after surgery (0 day vs. 3 months, *P* < 0.01), and decreased significantly more than 12 months with moderate-to-severe patients (0 day vs. 3 months, 3 months vs. 6 months, 6 months vs. 12 months, *P* < 0.01). The therapeutic effect of OSA of the mild group was significantly better compared with that of the moderate-severe group at 6 months (mean rank = 28.13 vs. 14.21, *P* < 0.001) and 12 m (mean rank = 26.75 vs. 15.52, *P* = 0.001). The APh results revealed that the pharyngeal volume of the two groups increased significantly between 0 day and 6 months after surgery (*P* < 0.01). The oropharyngeal junction (OPJ) area and the glottal area were increased significantly between 0 day and 6 m after surgery (*P* < 0.01).

**Conclusion:**

BS can relieve apnea and OSA symptoms among obese patients with OSA, especially in the early postoperative period. Moreover, OSA severity was closely associated with OPJ and glottal areas, rather than pharyngeal cavity volume.

## Background

Obstructive sleep apnea (OSA) is characterized by repeated episodes of apnea and hypopnea, hypercapnia, and sleep disruption. OSA is prevalent in the general population. At ≥ 5 events/h apnea–hypopnea index (AHI), the overall population prevalence ranged from 9 to 38% and was higher in men. It increased with increasing age and, in some elderly groups, was as high as 90% in men and 78% in women [1, 2]. OSA is closely associated with many diseases, including hypertension [3], coronary heart disease [4], arrhythmia [5], cerebrovascular disease [6], type 2 diabetes [7], non-alcoholic fatty liver disease [8], kidney damage [9], glaucoma [10], sexual dysfunction [11], as well as other organs and multiple system damage. OSA risk factors include obesity, male, advanced age, ethnicity, smoking, alcohol consumption, genetic predisposition, and abnormal upper respiratory tract anatomy [12]. Among them, obesity is one of the greatest risk factors of OSA [3]. Multiple cross-sectional studies have revealed a correlation between body mass index (BMI) and OSA risk [1, 13, 14]. Bariatric surgery (BS), also known as metabolic surgery, is one of the most effective obesity treatments and is the only effective long-term treatment for obese patients with OSA [15]. Longitudinal studies have shown that BS effectively reduces weight and significantly improves or resolves sleep apnea [2, 16]. However, such studies did not identify sleep apnea outcomes prospectively and did not meet all criteria for establishing causal relationship between BS and sleep apnea. Majority of previous studies have focused on surgery-induced weight loss, improvement of glycolipid metabolism disorder, and treatment of related cardiovascular and cerebrovascular diseases. However, whether BS causes structural changes to the upper respiratory tract and influences the occurrence of OSA is unclear. Acoustic pharyngometry (APh) is an FDA-approved, noninvasive diagnostic method for assessing sleep apnea [17]. APh measures the volume and cross-sectional area of the pharyngeal cavity using acoustic reflection. It is easy to use and non-invasive, making it suitable for early screening of sleep apnea [18]. Here, we used APh to examine the upper respiratory tract structure of obese patients with OSA before and after BS to determine whether BS alleviates OSA by improving the pharyngeal structure.

## Materials and methods

### Participants and criteria

Obese patients with OSA admitted to the Departments of Otolaryngology and General Surgery at our hospital from May 2018 to May 2020 were recruited into the study. The inclusion and exclusion criteria were based on the guidelines for surgical treatment of obesity and type 2 diabetes in China [19], and multidisciplinary guidelines for diagnostic testing of OSA in adults [20]. Patients who satisfied the following criteria were included in the study:

- Age = 18–65 years old.
- BMI ≥ 28 kg/m^2^ or waist circumference ≥ 90 cm for men and ≥ 85 cm for women.
- Diagnosed with OSA by polysomnography (PSG).
- Arterial blood gas values were within the normal range (PaCO_2_ < 45 mmHg, HCO_3_^−^ < 2 mmol/L) after non-invasive positive pressure ventilation (NPPV) therapy.

The exclusion criteria were as follows:

- Head and maxillofacial skeletal malformations or severe upper respiratory tract abnormalities.
- Poor physical conditions or other surgical contraindications.
- Lack of consent by participants.

Study indicators, including BMI, PSG, and APh results were evaluated preoperatively. NPPV treatment was performed before surgery for at least seven days to adjust blood oxygen concentrations to meet BS requirements. The same professional BS team at our hospital assisted with performing all operations. Afterward, the participants were requested to maintain reasonable diets and living habits according to the doctor's instructions, in addition to being reviewed regularly. Participants were followed-up for 12 months after BS and the aforementioned indicators were reexamined at 3, 6, and 12 months after BS.

Ethical approval for the present study was granted by the research ethics committee of The second hospital of Hebei Medical University (approval number 2018-R251). All methods were performed in accordance with the relevant guidelines and regulations. The health, rights, and privacy of participants were fully protected. Potential risks to participants were minimal and controllable.

### Demographic and anthropometric measurements

All participants filled in standardized questionnaires containing demographic information, such as sex, age, and education level. Data were carefully collected and recorded. Participant anthropometric measurements, such as height and weight were recorded and used to calculate BMI (BMI = weight/height^2^). Total weight loss (TWL) percentage = (initial weight − postoperative weight)/initial weight × l00%.

### Polysomnography tests

A 16-channel PSG monitoring device (Compumedics Ltd., Abbotsford, Victoria, Australia) was used to monitor participants for at least 7 h of night sleep. PSG recordings were used as the standard test method and scored by the same qualified sleep technologist based on the criteria published by the “Chinese Medical Doctors Association of Sleep Medicine Specialized Committee” [20] and the “American Academy of Sleep Medicine” [21]. “Apnea–hypopnea index (AHI)” was defined as the sum of apneas and hypopneas per hour of sleep. AHI > 5 was diagnosed as OSA. The therapeutic effect of OSA was categorized based on the changes in AHI before and after treatment as the standard as follows: “Cure” indicated AHI < 5 after treatment (denoted as 3 points); “Excellent” indicated AHI < 20 and a reduction ≥ 50% (denoted as 2 points); “Effective” indicated AHI reduction ≥ 50% (denoted as 1 point); “Ineffective” referred to a reduction in AHI of < 50% (denoted as 0 point). In addition to AHI indicators, the degree of improvement of subjective symptoms and variations in hypoxemia should be taken into account when determining efficacy.

### Acoustic pharyngometry

**Fig. 1:**
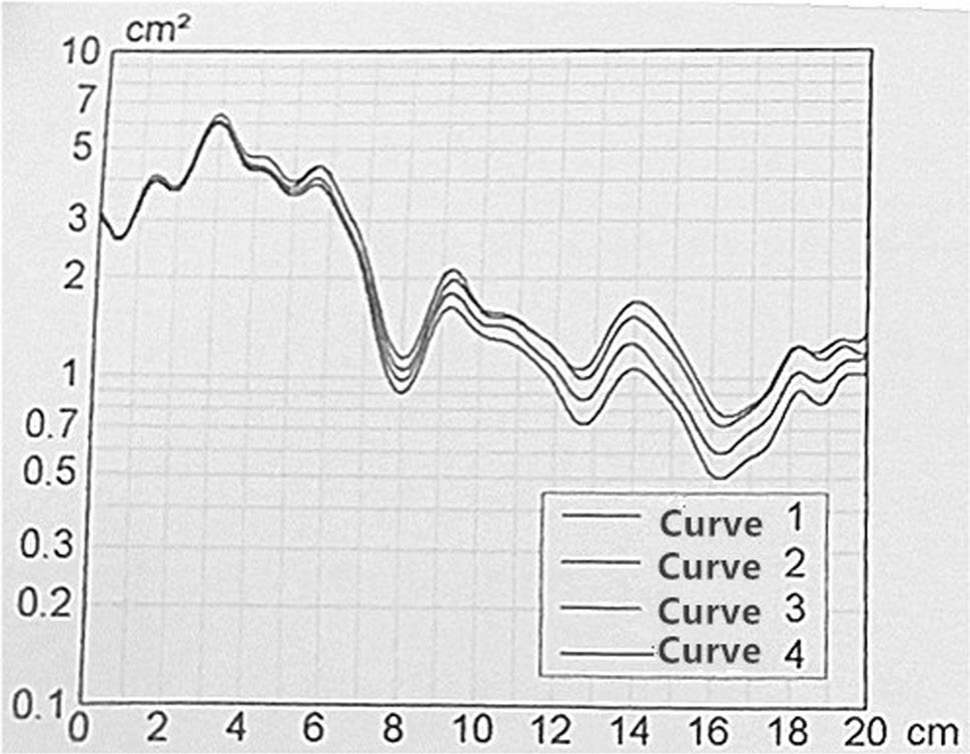
Acoustic pharyngometry (APh) curve(s)

**Fig. 2:**
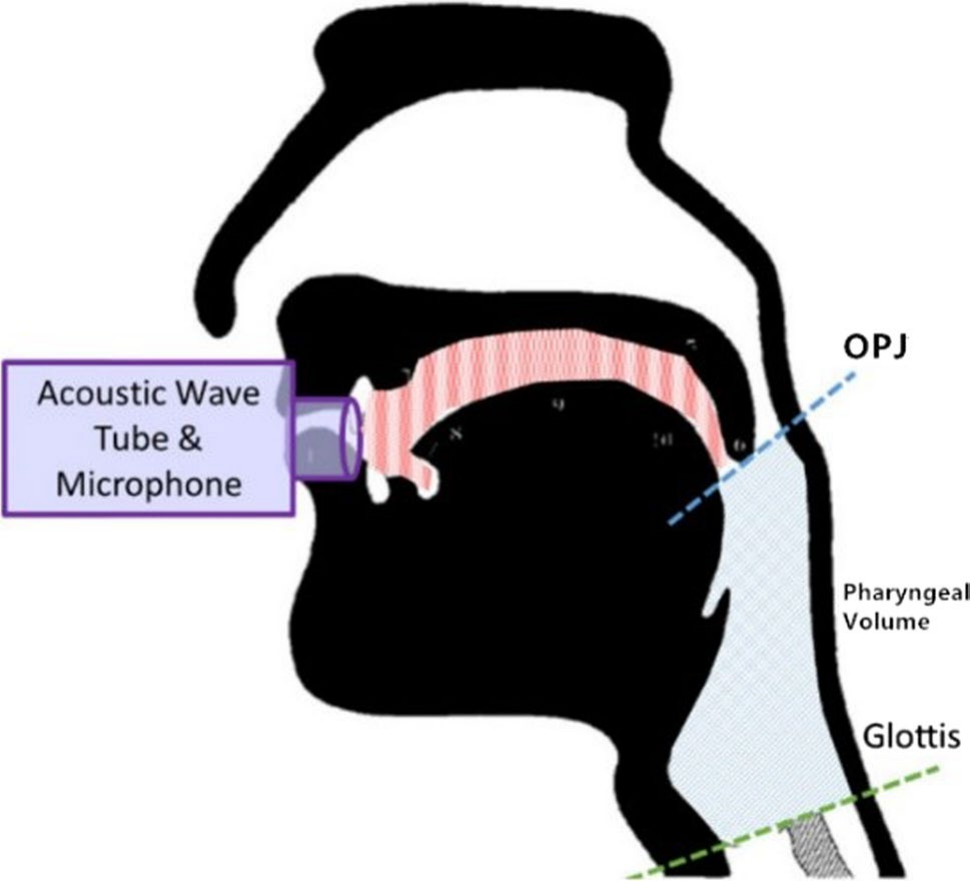
A schematic representation of acoustic pharyngometry (modified from Molfenter [17])

An acoustic rhinometer (GM Instruments Ltd., Irvine, North Ayrshire, UK) was used for APh. All operations were performed by the same experienced physician according to the standard operating procedures. The recorded results were plotted as graphs and area-distance curves obtained. Each operation was repeated four times and the average of the results was calculated; the repetition rate was maintained at 10% (Fig. 1). The measurement range was 0–20 cm and the observation range was approximately 7–17 cm (two distinct troughs corresponded to the oropharyngeal junction [OPJ] and the glottal area). The Y coordinate values corresponding to the two troughs represented the cross-sectional areas of the OPJ and glottis. In addition, the area under the curve between 7 and 17 cm was automatically calculated as the pharyngeal volume (Fig. 2). Based on our previous studies [22], cross-sectional areas of the OPJ and glottis, and the pharyngeal cavity volume were selected as APh indicators.

### Bariatric surgery

Laparoscopic sleeve gastrectomy was performed on all patients. The surgeon dissociated the gastric fundus and gastric greater curvature completely and used a 36-French bougie as gastric support. The starting point for gastric sleeve resection was 2–6 cm from the pylorus. The gastric fundus was completely removed and the whole cardia preserved. Finally, a sleeve-shaped stomach with a volume of approximately 60–80 mL was established. In the case of intraoperative hiatal hernia, treatment was administered at the same time. The wound was carefully sutured to minimize marginal bleeding.

### Statistical analyses

Data were analyzed using IBM SPSS Statistics 25 (IBM Corp., Armonk, NY, USA). Continuous variables were expressed as means ± standard deviation. Non-normally distributed data were logarithmically transformed before analyses. Comparisons of indicators between groups were performed using repeated measures Analysis of Variance if variances of differences between groups were equal or Welch’s test if variances were unequal to determine the impact of different interventions on the patients over time. Multiple comparisons were performed using Least Significance Difference test (for equal variances) or Games-Howell (for unequal variances). The point of therapeutic effect of OSA was based on ranked data; therefore, and Mann–Whitney U test was used to analyze variations between 6 and 12 months. *P* < 0.05 indicated statistical significance. Graphs were plotted using GraphPad Prism 6.0 (GraphPad Software Inc., La Jolla, CA, USA).

## Results

**Fig. 3:**
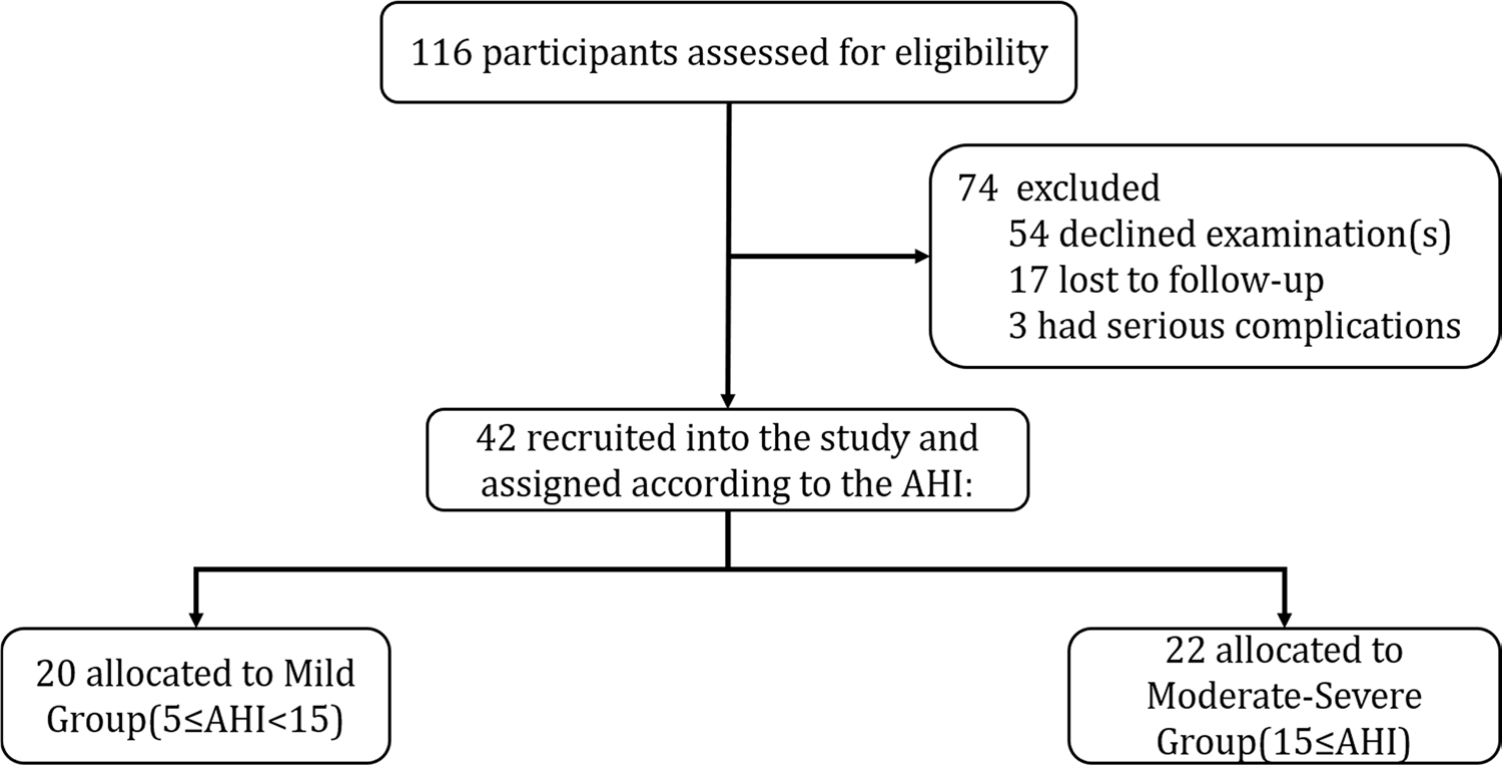
A flow chart illustrating the participants selection process

A total of 116 participants were evaluated for study eligibility. Among them, 54 were unwilling to undergo PSG examination after surgery due to financial constraints or other reasons, 17 participants were lost to follow-up, and three participants developed serious complications after BS. Therefore, 42 participants were finally recruited into the study and assigned to two groups based on their initial AHI values: mild group (5 ≤ AHI < 15) (n = 20) and moderate-severe group (15 ≤ AHI) (*n* = 22) (Fig. 3). No significant difference was observed between the groups in terms of demographic characteristics, such as age (*t*-test, *P* > 0.05) and gender (chi-square test, *P* > 0.05).

**Fig. 4:**
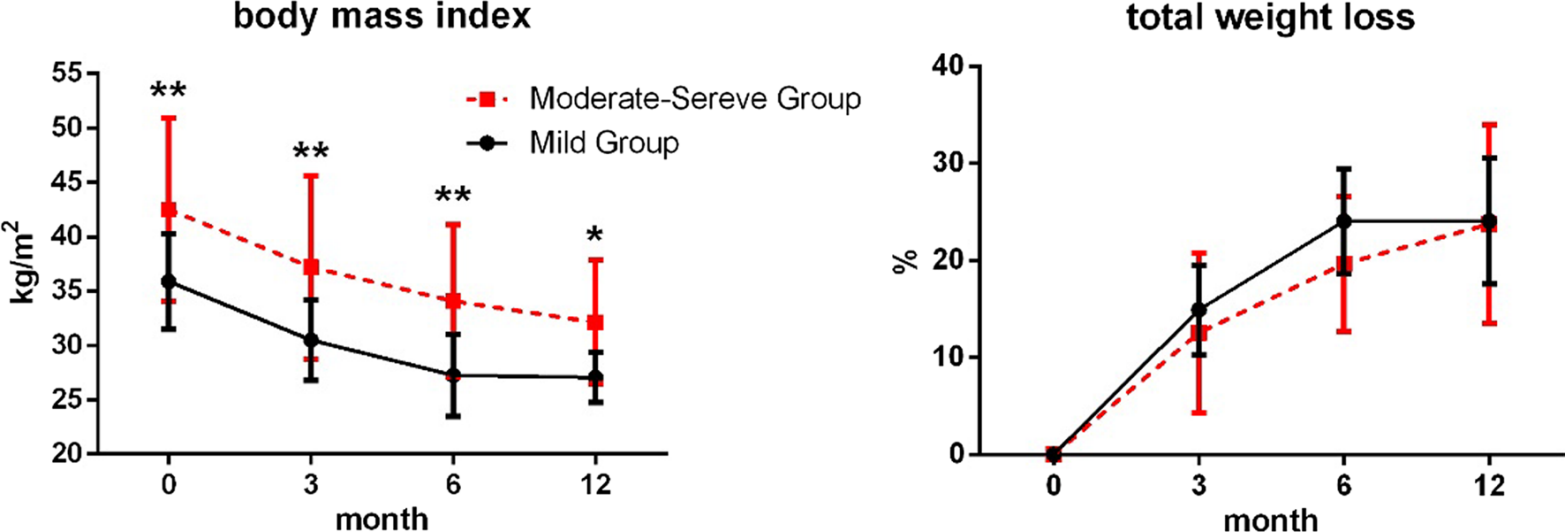
Variations in body mass index (BMI) and total weight loss (TWL) between the two groups of participants (mild and moderate-severe groups) at different time points. The black solid line represents the mild group while the red dashed line represents the moderate-severe group

**Fig. 5:**
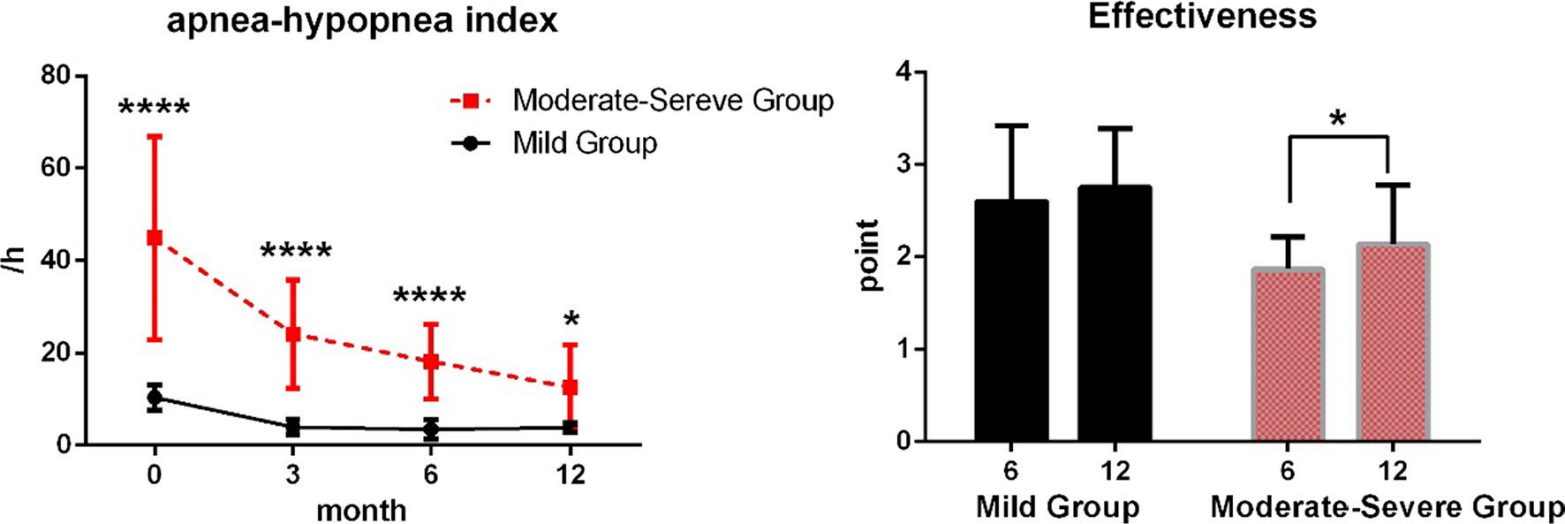
Variations in apnea–hypopnea index (AHI) in the two groups of participants (mild and moderate-severe groups) at various time points, and comparison of effectiveness. The black solid line represents the mild group while the red dashed line represents the moderate-severe group

**Fig. 6:**
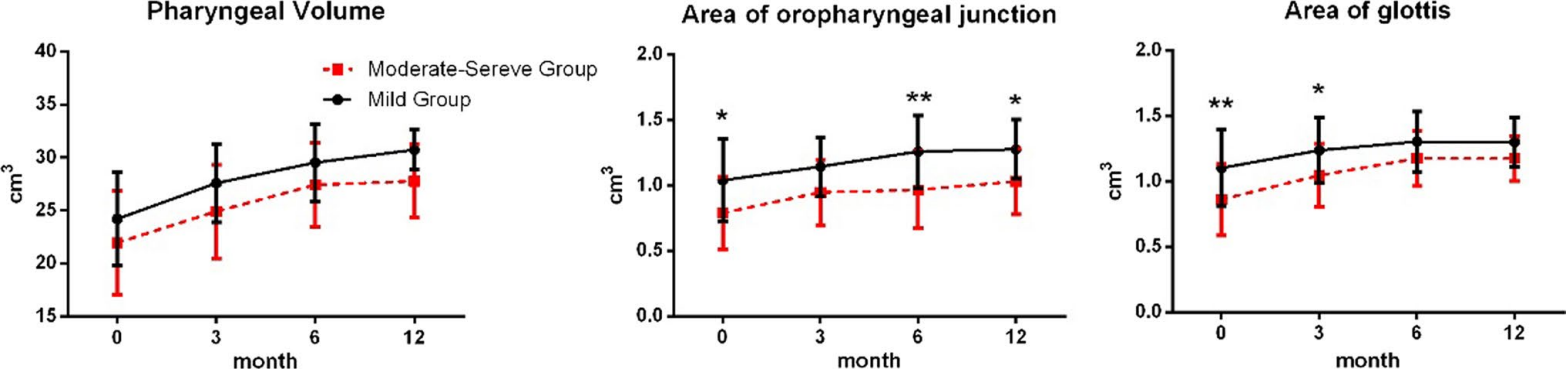
Variations in pharyngeal volumes, and the area of oropharyngeal junction and glottal of the two groups of participants at different time points. The black solid line represents the mild group while the red dashed line represents the moderate-severe group

The analyses results of the various indicators investigated among participants over time are presented in Figs. 4, 5, and 6. The results revealed that the indicators were significantly different between the groups and at various time points.

Data from all groups showed normal distribution as determined by the studentized residuals and Shapiro–Wilk test (*P* > 0.05). Furthermore, data had no outliers based on studentized residuals that exceeded ± 3 standard deviation criterion. Variance and covariance matrices of the dependent variables were equal after performing Mauchly's sphericity hypothesis test (*P* > 0.05) and the *P* value was < 0.001. The detailed results are as follows:

### Body mass index and total weight loss

According to the results, significant differences were observed in BMI at different time points (*F* = 128.1, *P* = 0.001) and between groups (*F* = 13.44, *P* < 0.001), but the interaction between time and grouping factor was not significant (*F* = 1.19, *P* = 0.315). The results of multiple comparisons showed that BMI of the mild group was significantly lower than that of the moderate-severe group at all time points (*P* = 0.002, *P* = 0.002, *P* = 0.001, and *P* = 0.026), and there were significant differences at each time point both in mild group and moderate to severe group (*P* < 0.01).

Significant differences were observed in TWL at various time points (*F* = 176.7, *P* < 0.001), while no significant difference was observed between groups (*F* = 1.697, *P* = 0.200) and the interaction between time and grouping factor (*F* = 1.558, *P* = 0.203). TWL in the two groups increased progressively from 0 days to 6 months (*P* < 0.001), and no significant difference was observed in TWL between 6 and 12 m (Fig. 4).

### Apnea–hypopnea index and therapeutic effect of OSA

Significant differences were observed in AHI at various time points (*F* = 83.09, *P* < 0.001), between groups (*F* = 53.39, *P* < 0.001), and the interaction between time and grouping factor (*F* = 33.86, *P* < 0.001). AHI was significantly higher in the mild group at 0 day than at 3 months, 6 months, and 12 months (*P* = 0.007, *P* = 0.003, and *P* = 0.006), and no significant difference was observed between the other time points. However, significant differences were observed in AHI in the moderate-severe group at each time point (*P* < 0.01). The data of AHI and lowest oxygen saturation are shown in Table 1

**Table 1:** The PSG data in two groups at 0 day, 6 months and 12 months

	0 day	6 months	12 months
Mild group			
AHI (/h)			
Mean ± SD	10.36 ± 2.72	3.45 ± 2.07	3.83 ± 1.02
Proportion (< 5: 5–15: 16–30: > 30)^a^	0: 20: 0: 0	15: 5: 0: 0	17: 3: 0: 0
Lowest oxygen saturation			
Mean ± SD	86.85 ± 3.66	95.75 ± 2.02	94.55 ± 2.44
Proportion (> 90: 89–85: 84–80: < 80)^b^	4: 10: 6: 0	20: 0: 0: 0	20: 0: 0: 0
Moderate-severe group			
AHI (/h)			
Mean ± SD	44.89 ± 21.46	18.16 ± 7.87	12.57 ± 9.38
Proportion (< 5: 5–15: 16–30: > 30)	0: 0: 6: 16	0: 10: 12: 0	5: 8: 8: 1
Lowest oxygen saturation			
Mean ± SD	75.72 ± 6.34	85.32 ± 6.57	88.01 ± 6.10
Proportion (> 90: 89–85: 84–80: < 80)	0: 3: 2: 17	6: 7: 5: 4	9: 7: 4: 2

^a^Corresponding to non-OSA, mild, moderate and severe OSA.

^b^Corresponding to non-hypoxemia, mild, moderate and severe hypoxemia

According to the results of Mann–Whitney *U* test, the therapeutic effect of OSA of the mild group was significantly higher than that of the moderate-severe group at 6 m (mean rank = 28.13 vs. 14.21, *P* < 0.001) and 12 m (mean rank = 26.75 vs. 15.52, *P* = 0.001). No significant difference was observed in effectiveness between 6 and 12 months in the mild group (mean rank = 19.55 vs. 21.45, *P* = 0.461), whereas a significant difference was observed in the moderate-severe group (mean rank = 19.41 vs. 25.59, *P* = 0.023), shown in Fig. 5.

### Acoustic pharyngometry indicators

No significant interactions were observed between time and grouping factors based on the three APh indicators (pharyngeal volume, OPJ area, and glottal area) (*F* = 0.523, *P* = 0.667; *F* = 1.454, *P* = 0.231; *F* = 2.449, *P* = 0.067).

Moreover, no significant difference was observed in the pharyngeal volume between the two groups at all time points (*P* = 0.237, *P* = 0.109, *P* = 0.299, and *P* = 0.061). Pharyngeal volume between the two groups increased progressively between 0 day and 6 months (*P* < 0.001), and no significant difference was observed in pharyngeal volume between 6 and 12 months (*P* = 0.134 and *P* = 0.906).

OPJ areas between the two groups of participants were significantly different at almost all time points (0 day: *P* = 0.011; 3 months: *P* = 0.071; 6 months: *P* = 0.002; 12 months: *P* = 0.013). OPJ area in the mild group increased progressively from 0 day to 6 months (*P* < 0.01), and no significant differences were observed in OPJ areas between 6 and 12 m (*P* = 0.948). However, OPJ area was significantly lower in the moderate-severe group at 0 day than at 3 months, 6 months, and 12 months (*P* < 0.001, *P* < 0.001, and *P* < 0.001), and no significant difference was observed in OPJ area among the other time points.

The glottal areas in the two groups of participants were significantly different at 0 day and 3 months (0 day: *P* = 0.004; 3 months: *P* = 0.037; 6 months: *P* = 0.275; 12 months: *P* = 0.290). Glottal area also exhibited an upward trend in the two groups. A significant increase in the glottal area was observed between 0 day and 3 months in the mild group (*P* = 0.002), with no difference being observed afterwards. A significant difference was observed in the glottal areas of the moderate-to-severe group between 0 day and 6 months (*P* < 0.01), whereas no significant difference was observed between 6 and 12 months (*P* = 0.999, shown in Fig. 6.

## Discussion

Obstructive sleep apnea (OSA) is a prevalent disorder characterized by partial or complete narrowing of the upper respiratory tract during sleep, in turn, resulting in repeated episodes of airflow cessation, oxygen desaturation, and sleep interruption. Multiple population-based studies have established that obesity is a major OSA risk factor. Over the last few decades, the prevalence of obesity has increased considerably across all age groups and ethnic backgrounds in developed countries. Similarly, sleep apnea rates have also increased [23]. Multiple active or passive weight loss approaches, including disease prevention, lifestyle and behavior changes, and pharmacotherapy, have been used to treat obesity-induced OSA. However, achieving weight loss through such methods is a challenge. Surgical approaches achieve long-lasting weight loss in severely obese individuals with BS and lasts more than 15 years [24]. The approach also resolves or relieves OSA symptoms in most morbidly obese individuals, enhances glycemic regulation, and decreases cardiovascular risk and obesity-related mortality [25, 26], in addition to exerting effects on all-cause mortality, cancer, and cardiovascular events [27].

Our results showed that participants with different OSA severity stages had different BMIs, and all exhibited significant decreases in BMI after BS. TWL was not significantly different between the two groups and increased progressively, which indicated weight loss, over the first six months after surgery, with no significant difference being observed thereafter. PSG results showed that the AHI values of participants in the mild group decreased during the early stage (0–3 months) and then remained unchanged; however, AHI values of the moderate-severe group decreased progressively until the end of the observation period (12 months). The therapeutic effect of OSA on the mild group was better than that of the moderate-severe group at 6 months and 12 months. These results suggests that BS can reduce excess weight gain in obese individuals, in turn, relieving symptoms such as sleep apnea caused by OSA to a certain extent. The participants in the mild group achieved remarkable therapeutic effect within a short time (at 3 months) after the operation, while the moderate-severe group required a longer time.

Several previous studies had demonstrated that BS can alleviate OSA symptoms in most morbidly obese individuals. The mechanisms by which BS affects sleep apnea are remain unclear. However, it is generally thought to be attributable to decreased fat accumulation [28] and insulin resistance [29]. Despite significant reductions in various indices, including BMI and AHI, the impact of weight loss on sleep architecture is complex and difficult to interpret, especially because participants remained obese even after surgery. However, otolaryngologists pay greater attention to pharyngeal structural changes before and after surgery as it is a key component of upper respiratory tract stenosis, although few studies have explored the aspect. The fat distribution at the base of the tongue does affect the structure of the pharyngeal cavity and the severity of OSA, but previous studies had shown that the percentage of fat in the tongue was not different between overweight participants and normal weight controls after adjusting for age, BMI, and gender [30]. Therefore, weight-loss after bariatric surgery will not significantly affect the distribution of fat at the base of the tongue. For obese patients with hypertrophy of tongue base, coblation-channelling of tongue (CCT) is still necessary. Therefore, bariatric surgery is more likely to alleviate respiratory obstruction in OSA by changing the structure of the pharyngeal cavity. The structure and pressure–area relationship (compliance curve) of the pharynx are considered an important factor in OSA pathogenesis [31]. The primary pathology associated with OSA involves soft tissue collapse in the oropharynx, resulting in ineffective or absent respiration. The Starling resistor model has been proposed as a model of explaining complications that occur during sleep in patients with OSA [32]. In the model, the oropharynx is considered a collapsible tube whose capacity to remain patent, partially patent, or closed, depends on the pressure in the upper and the lower respiratory tracts. The capacity of the oropharynx to remain patent is dependent on the airflow and pressure in the upper and lower respiratory tracts. Oropharyngeal tissue collapse may result from ineffective driving pressure, excessive negative or tissue pressure. Adipose tissue deposits around the respiratory tract among obese individuals reduces the upper respiratory tract dimensions, in turn, increasing airflow resistance [33]. Therefore, the pharynges of such individuals are narrower and more collapsible than those of normal individuals. Assuming constant airflow, respiratory tract narrowing results in more negative intrapulmonary pressure based on Bernoulli’s principle, which in turn, increases the likelihood of respiratory tract collapse. If the phenomenon occurs to a floppy respiratory tract either because of increased tissue compliance or decreased neuromuscular activation of the upper respiratory tract dilator muscles, OSA may develop. Therefore, evaluating pharyngeal cavity structure could provide novel methods and ideas for elucidating the underlying mechanisms and potential relationships between obesity and OSA.

APh is a novel, FDA-approved non-invasive method of sleep apnea diagnosis and treatment [17]. The technique measures pharyngeal cavity volume and cross-sectional area using acoustic reflection and is easy to use, with the capacity to identify upper respiratory tract obstruction sites in OSA. Kamal conducted an analysis of 350 normal volunteers using APh revealed that a definition for minimal pharyngeal area was required as a “gold standard” for examining individuals with OSA [34]. OPJ has also been suggested as a reliable indicator of OSA severity [35]. The effectiveness of APh in the determination of pharyngeal stenosis has been compared to those of computerized axial tomography (CT) [36] and magnetic resonance imaging (MRI) [37]. APh is inexpensive, rapid, does not involve exposure to radiation, and is easily reproducible relative to direct and fiberoptic visualization, roentgenographic cephalometry, fluoroscopy, CT, and MRI. We used APh to examine the pharyngeal structure considering that APh is a more accurate method of evaluating the pharyngeal cavity structure and diagnosing OSA. According to APh results, no significant difference was observed in the pharyngeal cavity volume among participants with different degrees of OSA; however, pharyngeal cavity volume increased with an increase in TWL. OPJ areas in the two groups of participants were significantly different at almost all time points, whereas the glottal areas were significantly different at 0 day and 3 months. The results suggest that the pharyngeal cavity structure was altered during the early stage after BS. However, most of the indicators, such as TWL, pharyngeal volume, and glottal area remained unchanged from 6 to 12 months.

With regard to the comparison of various indicators of participants with different OSA severity levels, we observed that BMI, and neck and waist circumferences increased with the severity of OSA. Notably, OPJ and glottal cross-sectional areas of participants in the mild group were significantly larger than those in the moderate-severe group, although pharyngeal volume did not differ significantly between the groups. The results suggest that the occurrence of airway obstruction is closely associated with minimum cross-sectional area of the pharyngeal cavity, with no correlation with the pharyngeal volume. Further analysis of the anatomical significance of the OPJ revealed that the cross-sectional area consisted of uvula, lymphoid tissues (such as tonsils and adenoids), and hyperplastic soft tissues. The glottis cross-sectional area is usually the narrowest region in the pharynx. These two areas could be associated with the upper respiratory tract narrowing and obstruction among obese individuals. Soft tissue accumulation and thickening are one of the major causes of the upper respiratory tract narrowing [38], which affects the tongue, uvula, tonsillar pillars, soft palate, blood vessels, lymphoid tissues, pharyngeal fat pads, muscles, and pharyngeal mucosa [39]. Tonsil enlargement and adenoid hypertrophy are the primary causes of OSA in children and are also commonly associated with OSA in adults. Furthermore, inflammation due to chronic trauma caused by tissue vibration during snoring [40] and gastroesophageal/laryngopharyngeal reflux [41] should be addressed. BS can inhibit chronic inflammation or stress response in the whole or parts of the body, leading to the gradual restoration of hypertrophic mucosal and lymphatic tissues to normal condition, which reduces the degree of upper airway muscle shortening, enlarge the minimum cross-sectional area of the pharyngeal cavity, and ultimate relief of sleep apnea symptoms.

## Conclusion

As well known, BS can reduce weight, alleviate fat accumulation in the neck and waist, alleviate apnea and OSA symptoms by adjusting metabolic balance, and regulate endocrine function. The present study has shown that the changes are mainly manifested during the early postoperative period (before six months). For patients with mild OSA, the results were highly significant within three months after surgery, while patients with moderate-to-severe OSA required more than 12 months to achieve significant outcomes. Regarding the structure of the pharynx, we established that OSA severity was closely associated with the cross-sectional area of OPJ and glottal areas but not with the pharyngeal cavity volume. For long-term treatment, the pharyngeal cavity structure in obese patients with moderate to severe OSA should be taken into consideration, except for weight loss, and other treatments such as uvulopalatopharyngoplasty should be recommended. Nevertheless, further studies are required to validate our findings, as well as elucidate the mechanisms underlying sleep apnea caused by obesity, which could facilitate the identification of novel individualized OSA therapies.

## Data Availability

All data presented here is included in the published article. Underlying data is available from the corresponding author upon reasonable request.

## Abbreviations

AHI: Apnea–hypopnea index
APh: Acoustic pharyngometry
BMI: Body mass index
BS: Bariatric surgery
CT: Computerized axial tomography
LSG: Laparoscopic sleeve gastrectomy
MRI: Magnetic resonance imaging
OPJ: Oropharyngeal junction
OSA: Obstructive sleep apnea
PSG: Polysomnography
TWL: Total weight loss
